# Same organ, divergent immunity: T-cell exhaustion versus stromal exclusion in hepatocellular carcinoma and intrahepatic cholangiocarcinoma

**DOI:** 10.3389/fimmu.2026.1944650

**Published:** 2026-10-16

**Authors:** YanXi Chen, Tao Peng, Hao Nie, Yan Hu

**Affiliations:** 1 Department of Oncology, The First Affiliated Hospital of Yangtze University, Jingzhou, Hubei, China; 2 Clinical Molecular Immunology Center, Department of Immunology, School of Medicine, Yangtze University, Jingzhou, China; 3 Department of Hepatobiliary Surgery, The First Affiliated Hospital of Yangtze University, The First People’s Hospital of Jingzhou, Jingzhou, China

**Keywords:** intrahepatic cholangiocarcinoma, hepatocellular carcinoma, immune checkpoint inhibitors, immune exclusion, T-cell exhaustion, tumor immune microenvironment

## Abstract

Although hepatocellular carcinoma (HCC) and intrahepatic cholangiocarcinoma (iCCA) arise in the same organ, they occupy distinct immune geographies within a shared barrier network. HCC is relatively enriched for inflamed yet dysfunctional CD8^+^ T-cell states, whereas iCCA is more often characterized by stromal- and myeloid-associated immune exclusion; both patterns can occur in either tumor, but differ in their relative predominance. Immune checkpoint blockade has reshaped the treatment landscape in primary liver cancer, but durable benefit remains limited and heterogeneous. Here, we place the T-cell landscape—not checkpoint expression alone—at the center of comparison, focusing on where tumor-reactive cells reside, the states they occupy, and the factors that constrain their reactivation. By integrating clinical and translational evidence across HCC and iCCA, and distinguishing throughout between mechanisms established in human tumors and those extrapolated from experimental models or from other malignancies, we propose that differences in the T-cell landscape help explain divergent therapeutic responses and provide a testable framework for biomarker-guided combination strategies. The key question is therefore not simply whether to release the checkpoint, but what each tumor’s T-cell ecosystem requires for effective reactivation.

## 1 Introduction

Primary liver cancer (PLC) ranks sixth in global cancer incidence and third in cancer-related mortality worldwide, with 5-year survival remaining below 20% (1, 2). Hepatocellular carcinoma (HCC) and intrahepatic cholangiocarcinoma (iCCA) share the hepatic niche but differ in their cellular origins and tumor microenvironments. HCC arises from hepatocytes in the context of chronic viral hepatitis, metabolic dysfunction, and cirrhosis. In contrast, iCCA arises from biliary epithelial cells in the context of chronic biliary inflammation, hepatolithiasis, or liver fluke infection. These distinct origins are reflected in their microenvironments: single-cell and spatial profiling that directly compares the two tumors has shown that HCC is more T-cell-infiltrated, whereas iCCA is more stroma-dominated and immune-excluded (3, 4).

The clinical landscape broadly mirrors this biology, although comparisons must be interpreted within the specific disease and treatment setting rather than by pooling across trials. In advanced HCC, first-line combination immunotherapy has become a standard treatment approach based on randomized phase III trials of atezolizumab plus bevacizumab and dual checkpoint blockade (STRIDE) (5, 6). These combinations, rather than single-agent PD-1 blockade, have produced objective response rates (ORRs) in the 20%–30% range. In contrast, nivolumab and pembrolizumab monotherapy, evaluated largely in previously treated patients in CheckMate-040 and KEYNOTE-224, respectively, have yielded response rates of approximately 15%–20% (7, 8). In biliary tract cancer (BTC), single-agent ICIs achieve objective responses in only approximately 5%–20% of patients, again primarily in pretreated, biomarker-unselected cohorts (9). (2) Adding immunotherapy to gemcitabine–cisplatin has yielded more modest gains in survival than in response depth, as noted in TOPAZ-1 (10) and KEYNOTE-966 (11). Notably, both trials enrolled patients with BTC overall rather than iCCA specifically, making direct extrapolation to iCCA indirect. Placed side by side, these response rates invite a comparison that the trials were not designed to support: the studies differ in tumor subtype, treatment line, chemotherapy backbone, comparator, and eligibility criteria. More informative than ranking one disease against the other is the shared pattern across these treatment settings: durable benefit remains confined to a minority of patients, and that minority is not adequately identified by the assays currently used to select patients. For iCCA specifically, the relevant treatment effect has not yet been established on its own terms. Both first-line BTC trials enrolled patients with tumors arising throughout the biliary tract, with iCCA accounting for approximately one-half to three-fifths of participants, while subsite analyses were exploratory rather than powered for definitive conclusions. Thus, the direction of benefit in iCCA is reassuring, but its magnitude remains largely inferred from the broader BTC population. The more relevant implication is therefore a within-trial rather than a between-trial observation: within individual cohorts, response appears to correlate less consistently with the extent of PD-L1 expression than with the location and functional state of tumor-infiltrating T cells (12, 13). Single-cell RNA sequencing (scRNA-seq) and spatially resolved transcriptomic approaches, including Visium and MERSCOPE, together with multiplexed proteomic profiling, have made this spatial logic accessible (13–15).

This framework should be viewed as a working model rather than a binary classification. HCC tends to exhibit intratumoral T-cell dysfunction with variable degrees of immune exclusion, whereas iCCA more frequently shows stromal- and myeloid-mediated exclusion. Nevertheless, substantial heterogeneity within and between tumors blurs these differences (16, 17) (**Table 1**; detailed classifications are provided in **Supplementary Table S1**). This framework provides the central axis of this review (**Figure 1**). Accordingly, we dissect the physical, cellular, and metabolic circuits that shape the distinct T-cell landscapes of HCC and iCCA and then consider which combination strategies logically follow from these differences.

**Table 1:** A conceptual alignment of HCC and iCCA immune phenotypes (18–20).

Immune phenotype	HCC— predominant pattern	iCCA— predominant pattern	Nature of the difference
Immune-infiltrated	Comparatively common; T-cell–rich lesions with active interferon signaling and PD-1/PD-L1 engagement, though effectors are frequently dysfunctional	Present but less common (the inflamed/immune-classical class); adaptive immunity and higher T-cell content in a minority of tumors	HCC more often sustains an inflamed—if exhausted—compartment; a genuinely inflamed iCCA subset exists but is the exception
Immune-altered (immunosuppressive)	Intermediate lesions with partial infiltration and mixed suppressive features	The larger share of iCCA; activated desmoplastic stroma and myeloid-dominated suppression restrict effector access	In iCCA the “altered” state is driven chiefly by stroma and myeloid cells rather than by intrinsic T-cell exhaustion
Immune-excluded	A defined subset, associated with WNT/β-catenin (*CTNNB1*) activation and low infiltration	A defined subset with a near-complete immune desert and marginal T-cell sequestration	Both tumors converge on WNT/β-catenin at the excluded pole, but iCCA more often reaches a true desert

Key point: This table aligns independently derived immune classifications of HCC and iCCA along a shared conceptual axis to aid comparison; it is a working model, not a unified taxonomy. The two classifications originate from different cohorts, clustering algorithms, and thresholds, so subtype frequencies are not directly comparable across tumor types and are therefore reported for each classification in **Supplementary Table S1** rather than side by side here. Source classifications: HCC (18, 19); iCCA (STIM) (20). HCC, hepatocellular carcinoma; iCCA, intrahepatic cholangiocarcinoma.

**Figure 1:**
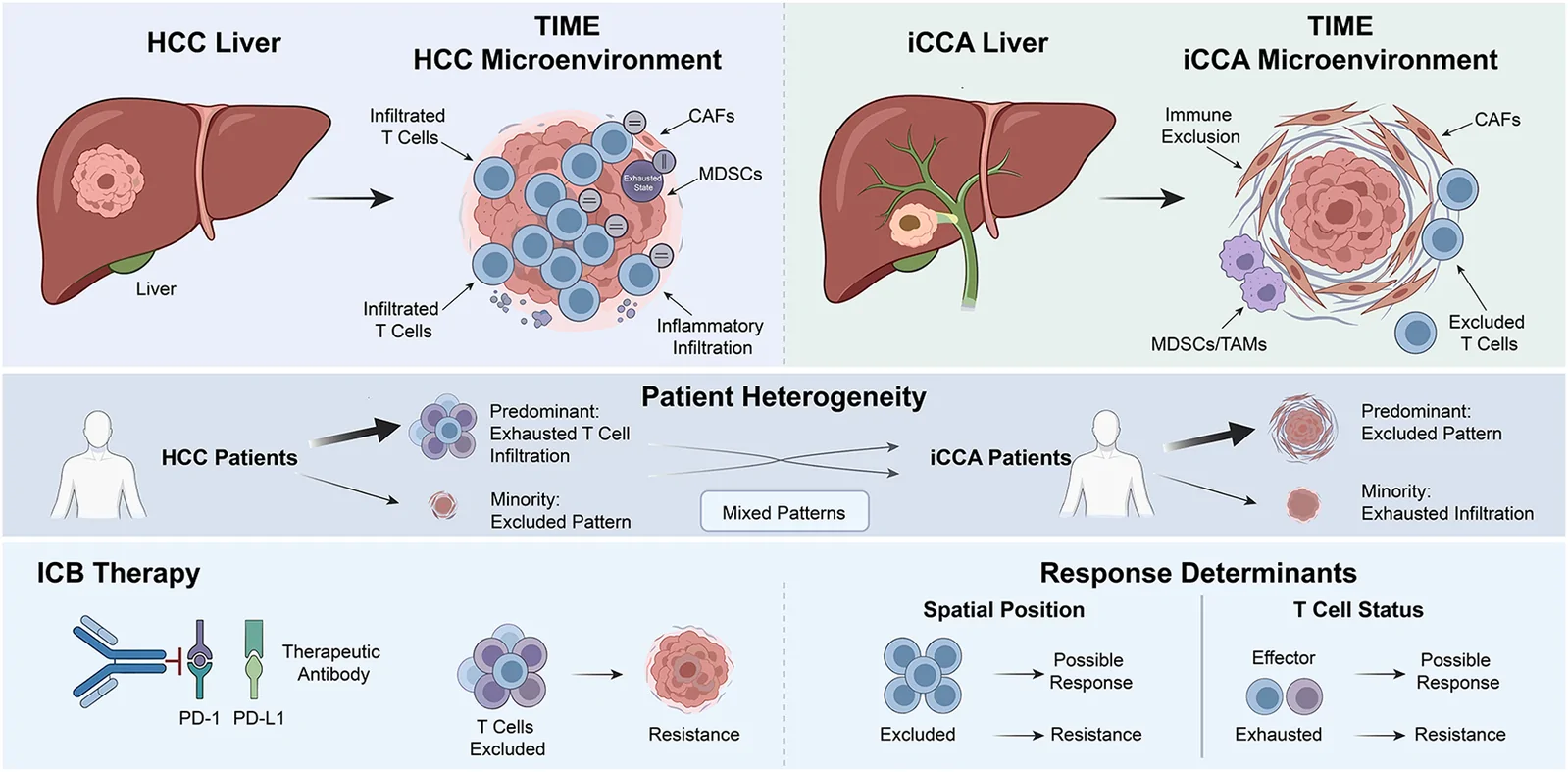
Divergent tumor immune microenvironments of HCC and iCCA and determinants of ICB response. While HCC predominantly features inflammatory infiltration with exhausted T cells, iCCA tends towards stroma- and myeloid-mediated immune exclusion. The figure illustrates significant patient heterogeneity, with both patterns seen in both cancer types. Crucially, clinical response to immune checkpoint blockade (ICB) therapy is shown to be dictated by T-cell spatial positioning (infiltrated vs. excluded) and functional status (effector vs. exhausted), rather than by simple PD-L1 expression.

## 2 Aberrant biliary repair sets the iCCA immune baseline

To understand why iCCA may be relatively resistant to therapy, it is important to consider the tissue context in which the tumor emerges. HCC often develops through repeated cycles of hepatocyte necrosis and regeneration within a cirrhotic liver (21). In contrast, iCCA more commonly arises in the setting of chronic biliary injury, cholestasis, and dysregulated ductular repair, with desmoplastic remodeling often, but not invariably, emerging early (22). Importantly, this background is heterogeneous: small-duct and large-duct iCCA can arise from distinct anatomical and etiological contexts, and a desmoplastic baseline should therefore be viewed as a frequent tendency rather than a universal feature. From this perspective, iCCA may represent not simply unchecked cellular proliferation, but the malignant co-option of a tissue-repair program that can establish a fibrotic, tumor-supportive microenvironment before the cancer is fully established.

The molecular wiring of this repair response provides a mechanistic bridge between tissue injury and malignant transformation, although much of the evidence remains model-dependent. In a mouse model of biliary injury, the IL-33–ILC2–IL-13 axis promoted cholangiocyte proliferation and epithelial repair; when this circuit was activated in AKT/YAP-primed bile ducts, however, it drove cholangiocarcinoma formation (23). Similarly, experimental coactivation of Notch and AKT reprogrammed hepatocytes towards a biliary phenotype that gave rise to rapidly progressive iCCA (24). In an extrahepatic biliary model, tumorigenesis likewise required coordinated Kras–AKT and Notch activation through mTORC1, rather than Notch activation alone (25). Together, these findings suggest that repair-associated lineage plasticity can become oncogenic when coupled to permissive signaling states, although whether this mechanism operates broadly in human iCCA remains unresolved.

This coupling between injury, repair, and oncogenic signaling may also shape the immune landscape through the progressive remodeling of the surrounding stroma. Chronic inflammation and fibrosis are associated with the accumulation of cancer-associated fibroblasts (CAFs), which can constitute a prominent stromal population (26–28). CAFs deposit and remodel the extracellular matrix (ECM), including through cross-linking mediated by the lysyl oxidase family (LOX and LOXL1–4) (29). The resulting increase in matrix stiffness can promote metabolic adaptation in both tumor and stromal cells (30) while creating a physical and biochemical barrier to immune-cell trafficking into tumor nests (31). Importantly, this barrier is not necessarily fixed. In cholangiocarcinoma models, inhibition of LOX-family activity reduces collagen cross-linking and matrix stiffness, facilitates intratumoral T-cell migration, and can enhance the efficacy of checkpoint blockade in solid-tumor models, including a cholangiocarcinoma model (32). In orthotopic and spontaneous iCCA models, selective pan-LOX inhibition with PXS-5505 reverses vascular compression and, when combined with chemotherapy, partially reprograms local antitumor immunity (33). Although these findings constitute mechanistic proof of concept rather than evidence of causality in patients, they support a broader view of fibrosis as an active determinant of tumor biology rather than a passive consequence of tumor growth. The resulting microenvironment can therefore become both physically restrictive and immunologically suppressive, with features of immune exclusion and, in some settings, relatively low immune-cell abundance (34, 35).

## 3 Spatial and functional splits in the PLC T cell landscape

### 3.1 Topology, not headcount, governs exclusion

For decades, the immune microenvironment was characterized largely by counting tumor-infiltrating lymphocytes. Spatial transcriptomics and multiplex imaging have shifted the focus from immune-cell abundance alone to spatial organization: lymphocyte density remains prognostically informative, but intratumoral localization, cellular neighborhoods, and proximity to malignant cells or suppressive myeloid niches provide additional biological and clinical information (36–38). Thus, a tumor can be lymphocyte-rich yet remain functionally immune-excluded if these cells fail to access or engage malignant-cell nests (**Figure 2**).

**Figure 2:**
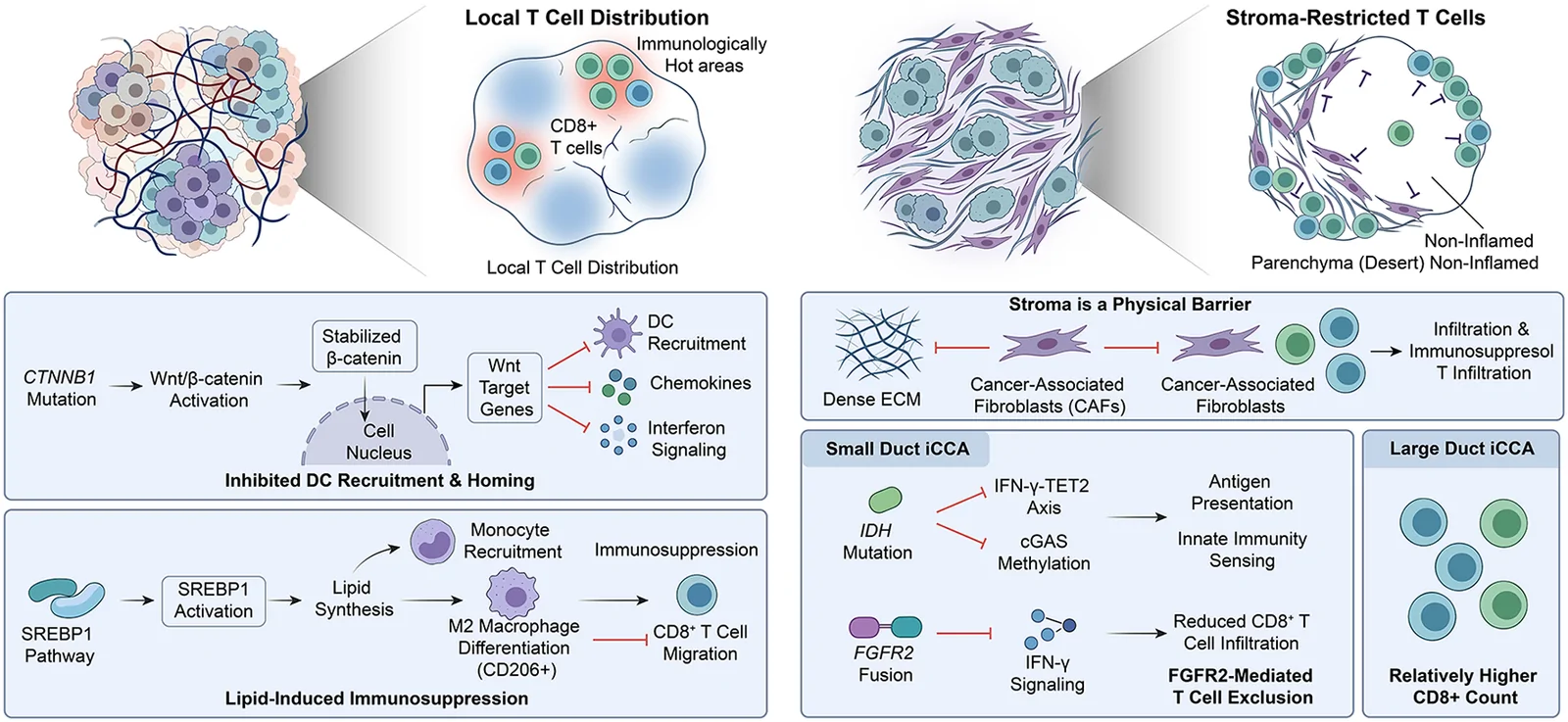
Distinct mechanisms of T-cell exclusion in HCC and iCCA. (Left) In HCC, CTNNB1 mutation activates the Wnt/β-catenin pathway to inhibit DC recruitment, chemokine signaling, and IFN signaling. Additionally, SREBP1-mediated lipid synthesis promotes the differentiation of immunosuppressive CD206^+^ macrophages and inhibits CD8^+^ T-cell migration, resulting in patchy, heterogeneous T-cell exclusion. (Right) In iCCA, a dense ECM creates a physical barrier, leading to diffuse, stroma-dominated T-cell exclusion. Small-duct iCCA further employs genetic mechanisms of immunosuppression: IDH mutations suppress antigen presentation and innate immune sensing, and *FGFR2* fusion reduces IFN-γ signaling and CD8^+^ T-cell infiltration. In contrast, large-duct iCCA features a relatively higher CD8^+^ count.

HCC and iCCA exhibit distinct, although overlapping, patterns within this spatial framework. In HCC, immune exclusion appears more often patchy than regionally pervasive in the settings studied and may be driven, at least in part, by tumor-intrinsic programs. Wnt/β-catenin signaling provides one of the best-supported examples: its activation impairs dendritic-cell recruitment and suppresses the chemokine and interferon programs required for effective T-cell infiltration, producing immune-excluded tumor nodules and reduced sensitivity to PD-1 blockade in preclinical models (39). Importantly, this phenotype appears to define a molecular subset rather than all non-inflamed HCCs, and the degree of exclusion may vary according to the signaling strength of individual *CTNNB1* mutations (40). Consistent with a causal role, β-catenin silencing can restore interferon signaling and T-cell recruitment in preclinical HCC models (41). A parallel, metabolic route involves tumor-cell SREBP1, whose lipid-biosynthetic program is associated with monocyte recruitment and can promote an immunosuppressive, CD206-expressing macrophage phenotype, accompanied by reduced CD8^+^ T-cell migration in preclinical coculture models (42). Together, these findings suggest that, in the contexts examined, HCC immune exclusion is spatially heterogeneous and can arise through distinct tumor-intrinsic and metabolic mechanisms. These pathways therefore provide a rationale for therapeutic targeting in selected molecular subsets, although whether such strategies can reliably reverse immune exclusion and improve treatment response in patients remains to be established.

iCCA shifts the same problem in scale: immune exclusion is often less patchy and more pervasive. Although far from universal, stromal exclusion is a predominant feature, with dense desmoplastic stroma imposing an immune-cold architecture across multiple molecular subtypes (**Table 1**). In non-inflamed tumors, parenchymal effector cells are sparse, whereas Tregs and macrophages may remain spatially compartmentalized (20). Even when CD8^+^ T cells are present, they frequently accumulate at the tumor margin or along the stroma–parenchyma interface rather than penetrating tumor nests (43, 44). Checkpoint expression is similarly spatially heterogeneous, with inhibitory programs detected within both the sclerotic tumor compartment and the tumor–stroma interface (45). The reported proportions vary according to cohort composition and phenotype definitions; across several cohorts, the majority of tumors have been classified as non-inflamed, a substantial minority as complete immune deserts, and only a smaller fraction as tumors with genuine parenchymal T-cell residency (16, 45).

Genotype may contribute to this architecture but does not fully explain it. Small-duct iCCA is enriched for *IDH* mutations and *FGFR2* fusions (46, 47). In model systems, mutant IDH has been reported to impair antigen presentation and innate sensing through DNA hypermethylation, including suppression of interferon-responsive genes through the IFN-γ–*TET2* axis and silencing of the dsDNA sensor *cGAS* (48, 49). These effects can be reversed by IDH inhibition in preclinical settings, but whether this translates into the restoration of antitumor immunity in patients remains uncertain; notably, ivosidenib-based combinations have shown limited clinical benefit (50). *FGFR2* fusions have likewise been associated with reduced CD8^+^ T-cell infiltration and a non-inflamed phenotype, potentially through suppression of IFN-γ signaling (51, 52). By contrast, large-duct iCCA has been associated with a different spatial profile, including relatively greater overall CD8^+^ T-cell infiltration (53). These observations remain population-level associations supported by preclinical mechanistic evidence rather than demonstrations of clinical causality.

The therapeutic challenge in iCCA is primarily architectural, although other factors also contribute. Reinvigorating exhausted T cells may have limited impact when physical and stromal barriers prevent their access to tumor nests. Conversely, strategies that remodel the stroma or promote tertiary lymphoid structures (TLS) may improve immune-cell recruitment, positioning, and local activation. These mechanisms are not mutually exclusive or unique to iCCA; rather, their relative contributions likely vary among tumors, with therapeutic resistance ultimately determined by the dominant constraints imposed by each tumor microenvironment.

### 3.2 Two roads to CD8^+^ exhaustion

Cytotoxic immunity ultimately depends on effector output, particularly granzyme B, perforin, and IFN-γ, and both HCC and iCCA can disrupt this output, although the dominant mechanisms may differ.

In HCC, chronic antigen exposure represents a prominent route to T-cell dysfunction, operating alongside vascular and myeloid exclusion. PD-1^Hi^ CD8^+^ T cells exhibit reduced cytokine and cytotoxic activity and frequently co-express TIM-3, a dysfunctional state associated with poor outcomes (54). Epigenetically, TOX helps stabilize this state by sustaining PD-1 expression and the exhausted T-cell program (55, 56). TIGIT defines an additional, functionally distinct layer: PD-1^+^TIGIT^+^ CD8^+^ T cells upregulate TOX and display impaired effector function, yet dual TIGIT/PD-1 blockade can restore proliferation and cytotoxicity, indicating that at least a subset retains pharmacologically recoverable function ex vivo (57). The critical clinical question, therefore, is not whether HCC T cells can be reactivated, but which populations remain reactivatable *in vivo*, a distinction that current biomarkers do not yet reliably resolve.

Together, these two tumors may constrain the same effector step through different predominant mechanisms: more often T-cell-intrinsic mechanisms in HCC and stromal or extrinsic mechanisms in iCCA. However, these mechanisms overlap, which may help explain why a shared checkpoint strategy could be less effective in iCCA unless the barrier is also addressed.

In iCCA, antigen-driven dysfunction may be compounded by extrinsic, stroma-imposed constraints. A CAF-rich, ECM-dense stroma is associated with the exclusion of CD8^+^ T cells from the epithelial compartment, often concentrating effector cells at the invasive margin and tumor–stroma interface, a pattern associated with poorer survival (44, 45). At the leading edge, SPP1^+^ macrophages colocalize with POSTN^+^ CAFs and endothelial cells, alongside margin-confined CD8^+^ T cells that exhibit low cytotoxicity and features of early exhaustion within a hypoxic niche (58). Chemokine-mediated retention of effector cells by antigen-presenting fibroblasts, described primarily in other desmoplastic tumors such as pancreatic cancer, may provide a related mechanism in iCCA, but this has not been directly demonstrated. Thus, CD8^+^ T-cell abundance alone may be misleading in iCCA: where T cells are located may matter as much as how many are present. This supports incorporating spatial localization and tumor–stroma proximity alongside density when evaluating patients for immunotherapy and suggests that therapeutic intervention may need to act upstream of the T cell itself.

One shared circuit may operate in both tumors, but its spatial wiring may differ. IFN-γ, produced by activated effector cells, can induce PD-L1 expression and thereby create a negative-feedback loop. In HCC, this response may occur predominantly on adjacent malignant hepatocytes, forming a relatively localized feedback shield; in iCCA, it may plausibly involve margin-resident myeloid cells that reinforce the tumor boundary. We present this contrast as an integrative inference from spatial and functional observations rather than as a single experimentally established pathway.

### 3.3 Tregs and the spatial logic of tolerance

CD4^+^ T cells can acquire cytotoxic programs, as demonstrated in a cholangiocarcinoma mouse model (59), but in human iCCA, the surrounding stromal architecture may constrain this potential. A recurrent feature reported in some studies is an MEOX1-associated hyperactivated Treg program, which has been linked to poorer survival (60) and may represent a more specific therapeutic target than the broad designation of “immunosuppression.” Genotype may further shape this landscape: co-occurring *TP53* and *KRAS* mutations have been associated with Treg accumulation and a non-inflamed, tumor-classical iCCA phenotype, although they do not define a uniformly excluded subtype (20). The spatial distribution of these Tregs, whether intratumoral or peritumoral, varies across studies, however, limiting their current utility as a robust spatial biomarker.

### 3.4 Rare functional subsets carry the reserve

When infiltrating lymphocyte numbers are limited, functional state and spatial position may become more informative than abundance alone. In reported iCCA cohorts, intratumoral effector CD8^+^ T cells have shown the clearest prognostic association (61). However, reactive markers validated in other tumor types may not carry the same meaning in iCCA; CD39^+^PD-1^+^ CD8^+^ T cells, which can identify tumor-reactive populations in other solid tumors (62), have instead been associated with an exhausted-like phenotype and poorer outcomes in iCCA (63). Such cross-tumor biomarkers may therefore misclassify the relevant functional compartment, underscoring the need for iCCA-specific definitions of context-cell reactivity.

Two candidate populations may be particularly informative. The first, TCF1^+^ precursor-exhausted (Tpex) cells, was characterized in murine chronic viral infection and in transplantable tumor models, where these cells retain stem-like renewal capacity and mediate the response to vaccination and checkpoint blockade (64); an equivalent population has not been functionally demonstrated in human iCCA, and its presence there is currently inferred from phenotypic marker concordance. The second, CD69^+^CD103^+^ tissue-resident CD8^+^ T cells, has been described directly in human cholangiocarcinoma specimens, although that evidence is descriptive and derives from a single cohort (65). Together, these observations suggest a model in which PD-1 blockade may act less by broadly reversing terminal exhaustion than by mobilizing a small, spatially accessible reservoir of potentially recoverable effector cells. This interpretation remains provisional, however, because PD-1 reflects both activation and dysfunction.

## 4 What builds and holds the iCCA barrier

### 4.1 The desmoplastic stroma as an active interface

The iCCA stroma is not merely inert packing; rather, it forms a CAF-organized interface, with hepatic stellate cell–derived CAFs representing a prominent population whose abundance is associated with worse survival (66). Three interrelated layers appear particularly relevant (**Figure 3**). First, mechanics. iCCA reportedly upregulates all five lysyl oxidase isoforms, and pan-LOX inhibition in preclinical models reverses mechanical compression of the tumor vasculature, improving drug penetration and antitumor T-cell responses (33). These findings provide mechanistic evidence that matrix stiffness can act as an active constraint rather than a passive scaffold. Elastography places iCCA lesions well above normal liver, in the tens of kilopascals depending on the modality and cohort (67, 68), although these are bulk, lesion-level measurements and should not be equated with the micron-scale mechanical cues sensed by individual cells. Aligned collagen offers a plausible contact-guided track that could direct T cells along the tumor margin, a mechanism demonstrated in other tumor types but not yet directly established in iCCA. Second, chemokine retention. Effector T cells may be retained in CAF-rich regions through the CXCL12–CXCR4 axis, which is directly established in pancreatic desmoplasia (69) and is biologically plausible, although not yet demonstrated, in iCCA. Third, CAF–myeloid crosstalk. iCCA CAFs have been reported to be a major source of CCL2, and FAP-driven STAT3–CCL2 signaling can recruit MDSCs (70), providing a myeloid-suppressive arm that may compound physical exclusion. This suggests that dense stroma may impede lymphocyte access while still permitting greater myeloid infiltration. These layers may be interlocking rather than independent: the same matrix that restricts T-cell access could facilitate the accumulation of myeloid suppressor cells. Thus, the stroma may function less as a collection of independent pathways than as an integrated, self-reinforcing barrier.

**Figure 3:**
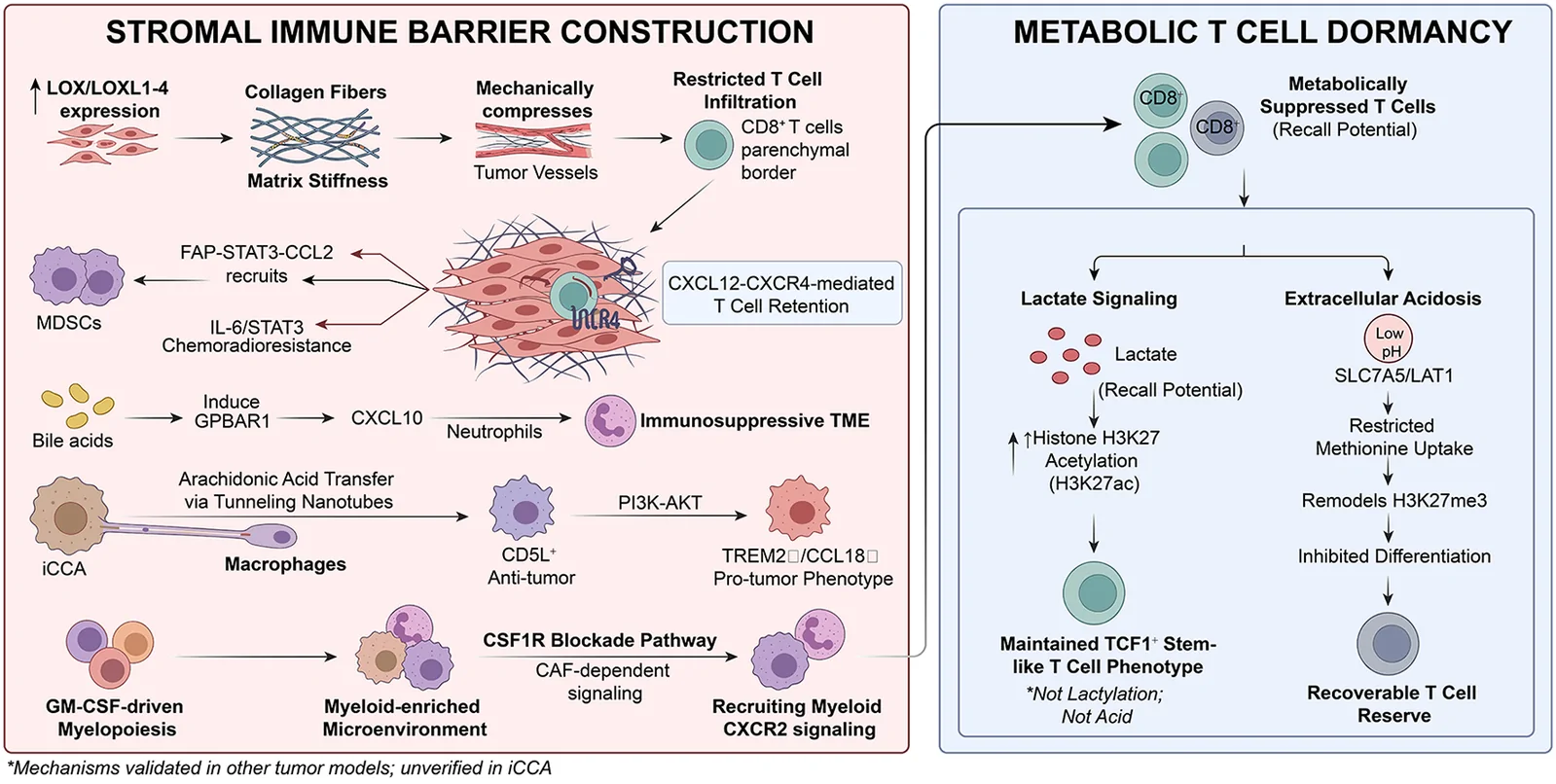
Multi-layered immune barrier construction and metabolic T-cell dormancy in iCCA. (Left) iCCA stroma actively constructs a multi-layered immune barrier. LOX enzymes-driven matrix stiffening restricts T-cell infiltration, while CAFs (via CXCL12-CXCR4, FAP-STAT3-CCL2, and IL-6/STAT3) retain T cells, recruit MDSCs, and mediate resistance. Bile acids trigger neutrophil recruitment via CAF GPBAR1-CXCL10 and drive protumor macrophage polarization via lipid transfer. GM-CSF causes myeloid enrichment, with CSF1R blockade inducing compensatory myeloid recruitment. (Right) Confined T cells adopt ‘adaptive dormancy’ (a metabolically suppressed state with recall potential). Two parallel metabolic mechanisms sustain this state (1): Lactate signaling maintains TCF1+ stem-like T cells via H3K27ac; and (2) extracellular acidosis downregulates SLC7A5, restricts methionine uptake, and remodels H3K27me3 to inhibit differentiation, preserving a recoverable reserve. These mechanisms have not been verified in iCCA.

Clinically, this interpretation is consistent with available data but remains incomplete. Non-inflamed, stroma-rich iCCA exhibits patterns of T-cell exclusion that may blunt ICI benefit (20); however, a direct patient-level association between matrix stiffness and ICI response has not been demonstrated. The barrier may also impose a chemotherapeutic cost through two routes: a soluble mechanism involving CAF-derived IL-6/STAT3 signaling, which has been implicated in gemcitabine resistance (71, 72), and a physical mechanism involving matrix compression and impaired drug penetration (33). Taken together, these findings support treating the stroma as an active and potentially actionable cotarget rather than as inert context. What remains lacking is patient-level evidence that matrix modulation can convert non-responders into responders; this translational gap, rather than the biological rationale itself, currently limits clinical application.

### 4.2 Bile acids reprogram the myeloid compartment

Compared with hepatocellular tumors, the biliary context may confer greater local prominence on bile-acid signaling in iCCA, although whether this reflects a genuinely greater bile-acid burden than in HCC remains unestablished. Two iCCA-focused mechanisms provide direct support for this model. The first is stromal: excess bile acids have been reported to activate *GPBAR1* on CAFs, including CXCL10, promoting EMT and metastasis, and recruiting neutrophils into an immunosuppressive niche that may also reduce pembrolizumab activity in preclinical models (73). The second involves metabolite transfer: iCCA cells transfer arachidonic acid to macrophages through tunneling nanotubes, a process associated with PI3K–AKT activation and a shift from antitumor CD5L^+^ macrophages toward protumor TREM2^+^/CCL18^+^ macrophages (74). The available direct evidence currently points more clearly to fibroblasts than to myeloid cells. The widely assumed “bile acid → TGR5 → suppressive macrophage” axis is largely extrapolated from non-iCCA models and remains unproven in iCCA, as do the c-Maf–IL-10 and arginase programs often attributed to iCCA tumor-associated macrophages (TAMs). An additional caveat concerns FXR, which is sometimes invoked as sustaining a protumor lipogenic state but has instead been associated with antitumor activity in human cholangiocarcinoma; its role may therefore not align with this proposed model. The most defensible interpretation is consequently narrower than the generic TAM-suppression model in which iCCA may establish an early stromal–myeloid suppressive field through at least one mechanistically supported bile-acid–stromal axis and one metabolite-transfer axis, whereas many other proposed components remain extrapolative.

### 4.3 A resilient multicellular network

Rather than acting independently, the suppressive populations in iCCA appear to be interconnected. CAFs have been reported to be a major source of CCL2, and FAP-driven STAT3–CCL2 signaling recruits MDSCs into the tumor (70), contributing to a myeloid-rich microenvironment. In both patients and autochthonous models, GM-CSF-driven myelopoiesis contributes to a predominantly myeloid infiltrate (75). Whether granulocytic MDSCs specifically constitute the majority, rather than myeloid cells more broadly, requires direct spatial and phenotypic quantification because granulocytic MDSCs and conventional neutrophils can be difficult to distinguish in tissue. The functional implication may be network redundancy, which could complicate single-node intervention. In other desmoplastic tumors, depletion of TAMs through CSF1R blockade can provoke CAF-dependent, CXCR2-sensitive recruitment of granulocytic MDSCs that repopulate the niche (76); whether the same compensatory rerouting occurs in iCCA remains untested. The implication is therefore conceptual but clinically relevant: a network with compensatory routes may blunt the effects of single-node targeting. A durable therapeutic strategy may consequently need to address multiple components of the myeloid circuit, potentially in combination, although the optimal targets, combinations, and treatment sequence remain unestablished.

### 4.4 Entrapment without terminal exhaustion? A testable hypothesis of a metabolically restrained T-cell reserve

One possibility is that not every tumor-reactive T-cell that fails to reach the tumor core is necessarily irreversibly lost. Studies of chronic infection and cancer have identified a TCF1^+^ precursor-exhausted population that retains self-renewal capacity, generates effector progeny, and remains responsive to PD-1 blockade (64, 77). These findings motivate, but do not establish, the hypothesis that iCCA may contain a metabolically restrained, recall-competent T-cell reserve. Here, “adaptive dormancy” is used only as conceptual shorthand for a putative, reversible state in which cells remain viable and capable of recall despite metabolic restraint; it does not imply canonical cell-cycle quiescence or an established TCF1^+^ precursor-exhausted identity. The metabolic rationale is plausible but largely extrapolated: nutrient limitation may increase AMPK activity and constrain mTOR-driven anabolism, whereas memory and precursor-exhausted cells tend to preserve oxidative respiration and spare respiratory capacity, together with more fused mitochondrial networks (78, 79). Whether this AMPK–mTOR configuration marks or helps maintain such a recoverable state in iCCA, however, remains untested (77). None of the observations supporting this rationale were obtained in iCCA, and the experiments proposed below to test it are likewise adapted from other tumor types; the section is therefore advanced as a falsifiable proposition rather than as a described feature of iCCA.

Two epigenetic inputs provide indirect mechanistic support for this hypothesis, although both are derived from models outside iCCA. Lactate, independently of its associated proton, inhibits histone deacetylases and increases H3K27 acetylation and histone lactylation at the *Tcf7* super-enhancer, thereby expanding stem-like TCF1^+^ CD8^+^ cells in tumor models (80). Extracellular acidosis may promote a similar phenotype through a distinct mechanism by downregulating *SLC7A5* (LAT1), restricting methionine uptake, and altering H3K27me3 at promoters of stemness-associated genes (81). Together, these observations support a two-phase hypothesis: metabolic stress may initially restrain differentiation while preserving a viable T-cell reserve, whereas subsequent relief of this stress may permit recall and effector recovery. Lactate and acidity should nevertheless be considered separate, context-dependent signals rather than components of a single “lactic-acid” mechanism.

Consistent with spatial studies that identified the iCCA leading edge as a stroma-rich barrier niche (58), a recoverable T-cell population, if present, would be expected to be enriched at fibrotic tumor–stroma interfaces, retain viability and TCF1-associated precursor features, and exhibit fewer transcriptional and epigenetic features of terminal exhaustion. TCR clonotype tracking, as used to demonstrate clonal revival and expansion of precursor-exhausted T cells during anti-PD-1 therapy in lung cancer (82), could reveal clonotypic continuity between these cells and more differentiated intratumoral progeny. Similarly, normalization of extracellular pH and/or restoration of nutrient availability, alone or in combination with antigenic restimulation or PD-1 blockade, could test whether these cells regain proliferative and effector functions ex vivo. Failure to recover after removal of these constraints would argue against the adaptive-dormancy model.

The translational implication is therefore conditional rather than established (**Table 2**). Mitochondrial pyruvate carrier (MPC) activity depends on the context: its inhibition can favor memory differentiation during ex vivo T-cell preparation, whereas intact MPC activity may support lactate oxidation and antitumor function within tumors (83). Dichloroacetate reduced tumor-derived lactic acid and improved T-cell proliferation, cytokine production, and viability in non-iCCA coculture experiments (84). By contrast, AZD3965-mediated MCT1 blockade improved CAR T-cell cytotoxicity and tumor control in B-cell malignancy models (85). These findings support the use of these agents as mechanistically distinct experimental perturbations of tumor lactate production and transport, respectively, rather than as validated approaches for reactivating iCCA T cells. Therefore, the resulting hypothesis is not that a distinct dormant T-cell subset is an established feature of iCCA, but that a subset of stromally entrapped, tumor-reactive cells may remain metabolically recoverable and could be neglected by strategies focused solely on terminal exhaustion.

**Table 2:** Priority mechanisms of T-cell dysfunction in iCCA: evidence and interpretation.

Immune phenotype	Priority mechanism	Core evidence	T-cell consequence	Critical interpretation
Immune- excluded	Desmoplastic and spatial barrier	myCAFs/POSTN, collagen-LOX remodeling, CXCL12-CXCR4 retention, and abnormal microvasculature; human spatial studies place many CD8^+^ cells at the invasive margin rather than in tumor nests (33, 58, 86, 87).	Poor penetration and margin sequestration	Best-supported iCCA-specific mechanism. Prioritize direct human spatial evidence; avoid treating every CAF product as independently causal.
Immune-altered (immunosuppressive)	Suppressive myeloid-Treg network	SPP1+/TREM2+ macrophages, PMN-MDSCs and hyperactivated Tregs converge through IL-10, TGF-beta, ARG1/iNOS and chemokine circuits (63, 70, 74, 75, 88–90).	Impaired priming, proliferation and cytotoxicity	Redundant circuits explain why single-node depletion can fail; combined stromal and myeloid targeting is a hypothesis, not yet a clinical standard.
	Metabolic and biliary niche	Hypoxia/acidosis, nutrient competition and IDO/TDO-kynurenine signaling coexist with bile-acid/*GPBAR1*-FXR effects (70, 73, 91).	Low mTOR/effector output; exhaustion or quiescence	Biologically plausible but uneven evidence: several pathways are preclinical or extrapolated from other tumors and should be labeled accordingly.
	Co-inhibitory signaling	PD-1/PD-L1, CTLA-4, TIM-3 and LAG-3 are enriched in selected niches, especially at the invasive front (58, 63, 92–96).	Dysfunction of the infiltrate that is already present	Checkpoint blockade cannot correct absent trafficking or weak priming by itself; expression is not equivalent to therapeutic dependence.
Reactive reserve	Tpex/TRM/TLS-associated reserve	TCF1+ precursor-exhausted cells, CD103^+^ tissue-residenT-cells, cDC1 and mature TLS identify a minority compartment with proliferative potential (63, 97–101).	Retained re-expansion capacity	A rational biomarker and therapeutic entry point, but prospective iCCA-specific validation remains limited.

### 4.5 Metabolism as the common current

Metabolic stress may affect both tumors but in different, partly overlapping configurations. HCC commonly exhibits aerobic glycolysis and altered glucose/lactate handling, although the extent of direct glucose competition with immune cells is model-dependent (102). In iCCA, desmoplastic stroma and abnormal vasculature are associated with impaired perfusion and hypoxia (103, 104). The immunological consequences are increasingly supported by direct profiling. In human iCCA specimens, single-cell and spatial profiling link *HIF1A*-driven hypoxia to a PPARγ–*CD36* program in APOE^+^ TAMs with immunosuppressive features and to CCL3–CCR5-associated Treg recruitment. The same analysis also identified CXCL13^+^ precursor-exhausted CD8^+^ T cells associated with preserved proliferative and cytotoxic capacity and better survival (105). Hypoxia may therefore function both as a suppressive driver and as a marker of a potentially reactivatable T-cell reserve.

Claims regarding the acidic milieu require caution. Lactate concentrations in the tens of millimolar range and extracellular pH values of approximately 6.0–6.5 have been reported for solid-tumor stroma in general rather than specifically measured in iCCA (106–108) and should therefore be regarded as contextual rather than iCCA-specific evidence. Nevertheless, lactate signaling in iCCA is supported at the tumor-cell level, where protein lactylation has been linked to ferroptosis resistance and PI3K-AKT signaling (109, 110). Histone lactylation has been reported to drive IGF2BP3-dependent, SPP1/CD44-mediated macrophage polarization in iCCA itself (90), and protein lactylation in iCCA tumor cells has been linked to ferroptosis resistance via ENO1 (111). By contrast, the corresponding immune-cell programs are better established in HCC than in iCCA: H3K18 lactylation in tumor-associated macrophages sustains an immunosuppressive phenotype that restrains CD8^+^ T-cell function (112), and MOESIN lactylation reinforces TGF-β signaling in regulatory T cells (113). Whether these macrophage- and Treg-directed programs operate similarly in iCCA immune cells remains under investigation.

Among the strongest iCCA-specific evidence are data on metabolic and lipid pathways. In *Clonorchis sinensis*–associated iCCA, profiled across 251 patients using single-cell, spatial, and metabolomic approaches, *FASN*-driven fatty-acid synthesis was associated with an immunosuppressive macrophage program and reduced T-cell cytotoxicity. *FASN* inhibition reversed these effects and improved anti-PD-1 efficacy in mouse models (114). AKT-hyperactivated iCCA has also been reported to sustain a pAKT–pPCK1–pLDHA axis that promotes ferroptosis resistance; this phenotype was relieved by simvastatin in preclinical models, improving chemoimmunotherapy. These findings are consistent with a proposed role for *PCK1* in restraining lactate accumulation and PI3K-AKT signaling (109, 110). A parallel pathway involving *MUC1*-stabilized FSP1 has likewise been implicated in iCCA. Viewed as a target landscape rather than as an established therapeutic framework, these metabolic dependencies are attractive but remain at an early stage. FSP1 inhibition has not been tested in combination with checkpoint blockade in iCCA, and *FASN* inhibitors such as denifanstat/TVB-2640 remain investigational agents developed in other oncology and metabolic-disease settings rather than established iCCA therapies (115, 116). The evidence therefore supports a cautious conclusion: metabolic cotargeting represents a substantiated preclinical rationale in iCCA, more firmly anchored in iCCA-specific data than generic metabolic extrapolation, but it is not yet a validated clinical strategy.

## 5 Regulatory dimensions unique to iCCA

### 5.1 Extracellular vesicles

Extracellular vesicles (EVs) may extend iCCA-associated immune and stromal remodeling beyond the tumor cell, although the strongest evidence currently concerns effects on myeloid cells, NK cells, and fibroblasts rather than direct regulation of T cells. One of the most direct examples is macrophage-mediated: iCCA-derived exosomal miR-183-5p has been reported to suppress *PTEN* and increase AKT-driven PD-L1 expression in TAMs, which in turn is associated with reduced CD8^+^ T-cell activity and poorer survival. This represents an indirect, macrophage-mediated mechanism of T-cell suppression rather than direct T-cell anergy (89). CCA-derived EVs have also been reported to impair NK-cell adhesion and cytotoxicity (117) and to enhance fibroblast migration and matrix remodeling (118). In *Clonorchis sinensis*–associated disease, parasite-derived EVs deliver Csi-let-7a-5p to macrophages and activate *Socs1/Clec7a*–NF-κB signaling (119). These findings, however, do not yet support the broader causal chain sometimes inferred. Direct evidence that CAF-derived EVs induce T-cell anergy or chemoresistance in iCCA is lacking, and a TLR-dependent EV mechanism of immune exclusion has not been demonstrated. Because many studies remain cell-line-based or descriptive, and because differential ultracentrifugation alone does not establish exosomal origin (120), EV signaling should be considered an emerging layer of immune–stromal regulation rather than an established mechanism of immune exclusion. This distinction has practical implications: mechanistic interpretations should remain cautious, whereas EVs may have more immediate potential as circulating candidate biomarkers. Plasma exosomal miR-183-5p has been associated with post-resection outcomes, suggesting that its near-term value lies more in prognostic biomarker development than in therapeutic targeting, although prospective validation remains necessary.

### 5.2 Bile acid signaling as an immune checkpoint

Beyond its stromal and myeloid effects, bile-acid signaling can also act directly on T cells, supporting the concept of a hepatobiliary metabolic checkpoint that is relevant, although not unique, to iCCA. In human HCC and experimental liver tumors, tumor-enriched bile acids have been reported to impair CD8^+^ T-cell function through distinct mechanisms: the conjugated primary bile acid TCDCA suppresses mitochondrial respiration and increases oxidative stress, whereas the hydrophobic secondary bile acid LCA acts through ER stress. Increasing the availability of ursodeoxycholic acid (UDCA) counteracts both effects and improves tumor control in experimental models (121). Direct evidence from iCCA supports the recoverability component of this hypothesis: FXR is overexpressed in exhausted CD8^+^ TILs in human and murine iCCA and directly upregulates *Lag3*, whereas genetic ablation of FXR enhances effector function and expands TCF1^+^TOX^+^ precursor-exhausted (Tpex) cells. Pharmacological FXR inhibition with UDCA similarly alleviates T-cell exhaustion and enhances anti-PD-L1 activity in murine iCCA and ex vivo human iCCA tumor fragments (91). These findings strengthen the iCCA-specific mechanistic case; however, given the small cohorts and ex vivo endpoints, they should be considered preclinical and translational evidence rather than evidence of clinical efficacy. A parallel CD4^+^ T-cell mechanism, in which bile acids engage S1PR–STAT3 signaling to repress FOXP3 and promote a Th17-like Treg state, has been described in fibrosing cholangiopathies but remains of unknown relevance to iCCA (122). Directionality is therefore an important caveat: inhibition of T-cell-intrinsic FXR appears to favor anti-iCCA immunity in iCCA, whereas the effects of bile acids are molecule- and cell-type-dependent and can be opposing (123, 124). Thus, modulation of bile-acid signaling cannot be assumed to be uniformly beneficial. The mechanistic rationale in iCCA is now more directly supported, but what remains missing is prospective, biomarker-rich testing of whether UDCA or cell-selective, directionally defined bile-acid-receptor modulators can reactivate CD8^+^ T cells and improve the efficacy of checkpoint blockade in patients.

### 5.3 Liquid biopsy under desmoplastic constraints

Liquid biopsy in iCCA should be interpreted in the context of a tumor that is often densely desmoplastic and spatially heterogeneous with respect to immune infiltration, two features that have been established independently rather than causally linked in any single study. Multiregional profiling shows marked variation in immune infiltration among regions of the same tumor (45, 52), indicating that a single specimen may not fully capture the tumor’s immune architecture. A tissue core may therefore be spatially biased, whereas a blood draw provides a time-specific, shedding-weighted molecular mixture. This limitation may be particularly relevant in low-shedding, stroma-rich CCA, where plasma ctDNA sensitivity is limited (125). When tumor-derived signal is adequate, serial and tumor-informed ctDNA assays have shown potential for tracking molecular residual disease, treatment response, and emerging resistance (126–129). Bile-derived cfDNA may enrich tumor-derived DNA relative to plasma, although the supporting evidence comes largely from biliary-stricture cohorts sampled invasively rather than from typical mass-forming iCCA (130, 131). Integrating these circulating biomarkers with spatial immune profiling into multimodal models remains investigational (132). For an immune-focused interpretation, the practical implication is therefore limited but important: circulating biomarkers should be viewed as longitudinal complements to spatial immune analyses rather than as substitutes for them. They can track aspects of molecular disease over time but cannot, on their own, reveal where T cells are located within the tumor.

## 6 Clinical translation

### 6.1 Why monotherapy stalls

Single-agent checkpoint blockade has shown limited activity in PLC, and clinical trial experience supports this limitation. Nivolumab did not improve overall survival (OS) in advanced HCC, while single-agent pembrolizumab has achieved response rates of approximately 6%–13% in BTC (133, 134) (**Figure 4**). One explanation is structural: checkpoint blockade releases a single inhibitory axis within tumors shaped by multiple overlapping constraints. Single-cell and spatial atlases identify checkpoint-high, dysfunctional T cells alongside Treg- and myeloid-mediated suppression in both diseases (16, 60, 135). However, suppression is only half of the problem. On the activation side, defective MHC-I/antigen presentation and processing, together with inadequate dendritic-cell-dependent priming, can leave relatively few tumor-reactive CD8^+^ clones available for expansion following checkpoint blockade (13, 136, 137). This deficit may be at least as important as inhibitory pathways and, in immune-cold tumors, may represent a more proximal barrier to therapeutic response.

**Figure 4:**
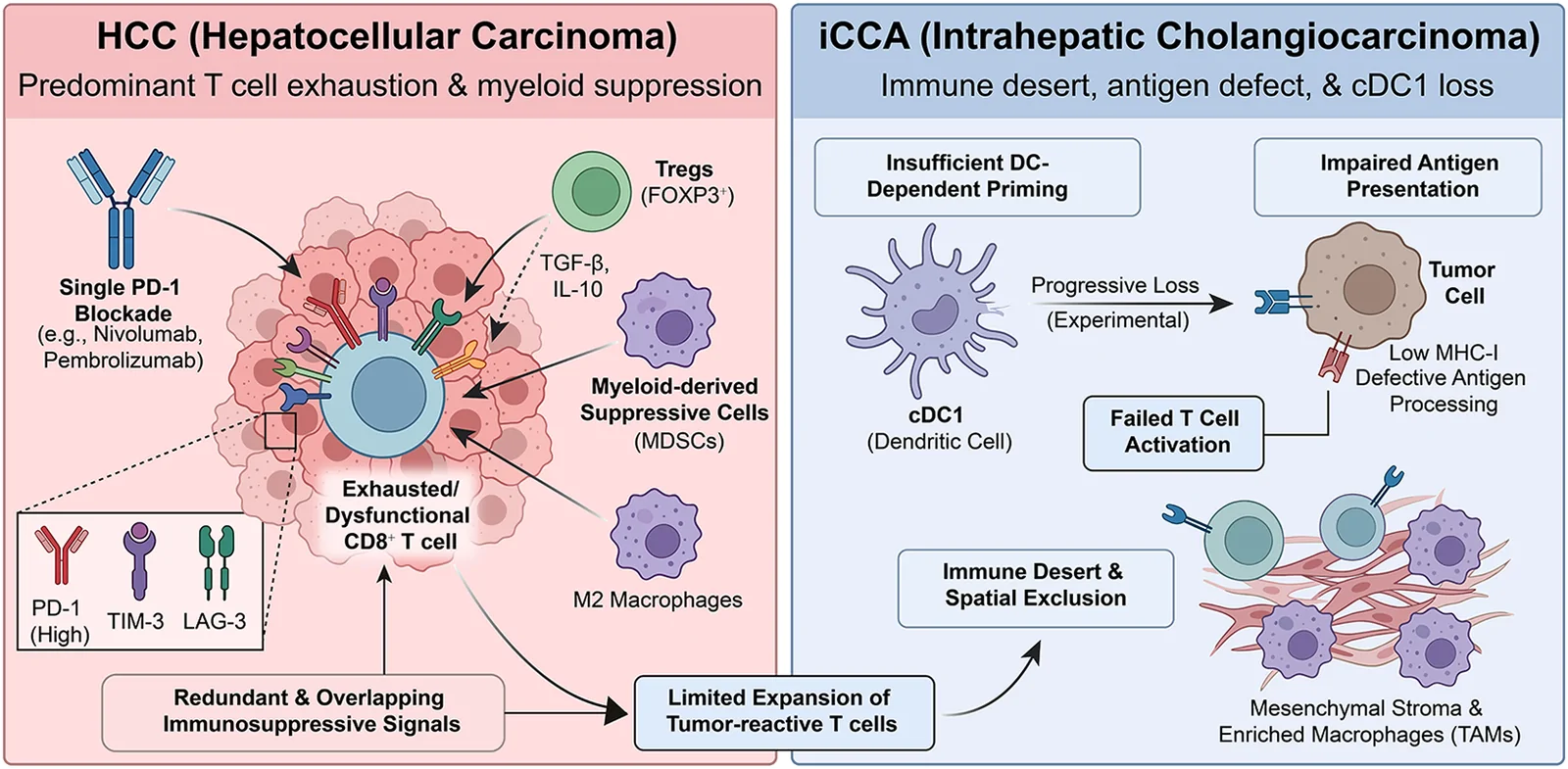
Mechanisms of limited PD-1/PD-L1 blockade efficacy in HCC and iCCA. Single-agent PD-1/PD-L1 blockade (e.g., nivolumab and pembrolizumab) faces distinct microenvironmental hurdles in liver cancers. In hepatocellular carcinoma (HCC), efficacy is heavily restricted by redundant immunosuppressive signals, characterized by highly exhausted T cells and active suppression by Tregs and myeloid-derived suppressive cells (MDSCs). Conversely, intrahepatic cholangiocarcinoma (iCCA) frequently presents as an immune desert with spatial exclusion, driven by a mesenchymal stroma, impaired antigen presentation (low MHC-I), and insufficient priming due to the progressive loss of cDC1 dendritic cells. Ultimately, these distinct pathways both result in limited expansion of tumor-reactive CD8^+^ T cells.

These failure modes—weak antigen presentation, spatial exclusion, and redundant suppression—are shared across HCC and iCCA, but their contributions are likely subtype-dependent rather than fixed. Direct HCC–iCCA comparisons provide an important corrective to the assumption that iCCA is simply “more myeloid-suppressed”. T-cell exhaustion, together with several myeloid-driven suppressive pathways, may in fact be more pronounced in HCC than in iCCA (138). iCCA is more consistently associated with immune-desert, mesenchymal, and macrophage-rich spatial states (16). Progressive loss of cDC1s with tumor progression has likewise been documented in experimental iCCA, although this phenomenon has not been established across human liver cancers (139). The therapeutic implication is consistent across both diseases: in both HCC and iCCA models, strategies that activate or recruit antigen-presenting cells while simultaneously blocking PD-1 have often outperformed PD-1 blockade alone in preclinical studies (96, 140). Releasing an inhibitory brake may achieve little when insufficient antigenic stimulation is available. Thus, these two deficits may be best addressed in a coordinated manner, although the optimal targets and treatment sequence remain unresolved.

### 6.2 Pairing checkpoint blockade with agonists

The randomized clinical record illustrates both the progress of checkpoint-based therapy and its current limitations. In unresectable HCC, the STRIDE regimen of tremelimumab plus durvalumab achieved a 5-year OS rate of 19.6% versus 9.4% with sorafenib (HR 0.76), providing a meaningful signal of long-term durability, although the 5-year survival rate remained below 20% (141). Adding anti-angiogenic therapy has not consistently improved OS. In COSMIC-312, cabozantinib plus atezolizumab improved PFS but not OS (16.5 vs 15.5 months; HR 0.98) (142), while single-agent cabozantinib produced an ORR of 4% in previously treated disease (143). In BTC, TOPAZ-1 established durvalumab plus gemcitabine–cisplatin as a first-line standard, although the survival benefit was incremental (median OS 12.9 vs 11.3 months; HR 0.74). Real-world PFS has been reported at approximately 8 months, and no clinically validated biomarker currently identifies patients most likely to benefit (144, 145). Overall, the clinical signal remains limited in magnitude: checkpoint-centered regimens produce durable benefit in a minority of patients with HCC and more modest gains in biliary cancer. These observations support exploration of additional immune inputs rather than assuming that intensification of single-axis checkpoint blockade alone will be sufficient.

Two investigational directions seek to provide such a second input, but their evidence remains preclinical and should not be overinterpreted. Costimulatory agonism involving OX40, 4-1BB, ICOS, or CD40 aims to provide positive signals that are not supplied by checkpoint blockade. In orthotopic HCC models, CD40 agonism can reprogram TREM2^+^ macrophages that otherwise limit the activity of GPC3-CAR-T cells in preclinical models, although the clinical CD40 agonist sotigalimab has so far been shown only to enhance CD8^+^ T-cell migration *in vitro*. These findings support the mechanistic rationale for combination therapy but not clinical efficacy (146). LAG-3, as an inhibitory receptor, belongs in this framework as a target for blockade rather than agonism. Innate-sensing agonism is similarly at an early stage. The acid-activated STING agonist MSA-2 has exhibited stage-dependent activity in murine iCCA, with advanced, PD-L1-high tumors requiring MSA-2 plus PD-L1 blockade for tumor control (147, 148). Other cGAS–STING agonist carriers have been evaluated in different tumor types or with different combination partners and therefore cannot be treated as direct evidence in iCCA. To date, none of these approaches has demonstrated efficacy in patients with iCCA or in randomized studies. They should therefore be viewed as rational strategies for clinical investigation against the desmoplastic barrier rather than as interventions already demonstrated to overcome it.

### 6.3 Subtype-specific remodeling

These tumors may warrant different initial therapeutic priorities but share overlapping vascular, myeloid, and fibrotic barriers whose relative contributions vary across molecular and immune subtypes. This distinction is therefore one of emphasis rather than one of mutually exclusive therapeutic prescriptions (**Figure 5**; **Table 3**). In HCC, angiogenesis-linked immunosuppression currently provides one of the more clinically anchored entry points. Analyses of GO30140/IMbrave150 specimens associated benefit from atezolizumab plus bevacizumab with pre-existing CD8^+^ immunity and suggested that VEGF blockade restrains Treg proliferation and myeloid inflammation (149). Single-cell profiling further associated durable responses with CXCL10^+^ macrophages and clonally expanded cytotoxic CD8^+^ T cells (150). Multitarget TKIs may act on overlapping axes. In serial baseline and 4-week HCC biopsies, lenvatinib treatment was associated with lower proportions of CD3^+^CD4^+^FOXP3^+^ cells among CD3^+^CD4^+^ T cells and CD68^+^CD163^+^ macrophages among CD68^+^ macrophages (151). In a separate neoadjuvant study, cabozantinib, targeting VEGFR2, MET, and AXL, combined with nivolumab achieved a major pathological response in 5 of 12 resected HCC tumors (42%), with responders enriched for tertiary lymphoid structures and organized B-cell niches (152). Locoregional combinations further suggest that benefit may be greater when checkpoint blockade is paired with anti-angiogenic therapy. LEAP-012 and EMERALD-1 improved PFS, whereas durvalumab plus TACE without bevacizumab was not associated with a similar PFS improvement (153, 154). Across the pooled NeoHCC analysis, the neoadjuvant MPR benchmark was 32%; therefore, the 42% response observed in the cabozantinib–nivolumab study should be interpreted as a selected early-phase result rather than a generalizable response rate (155).

**Figure 5:**
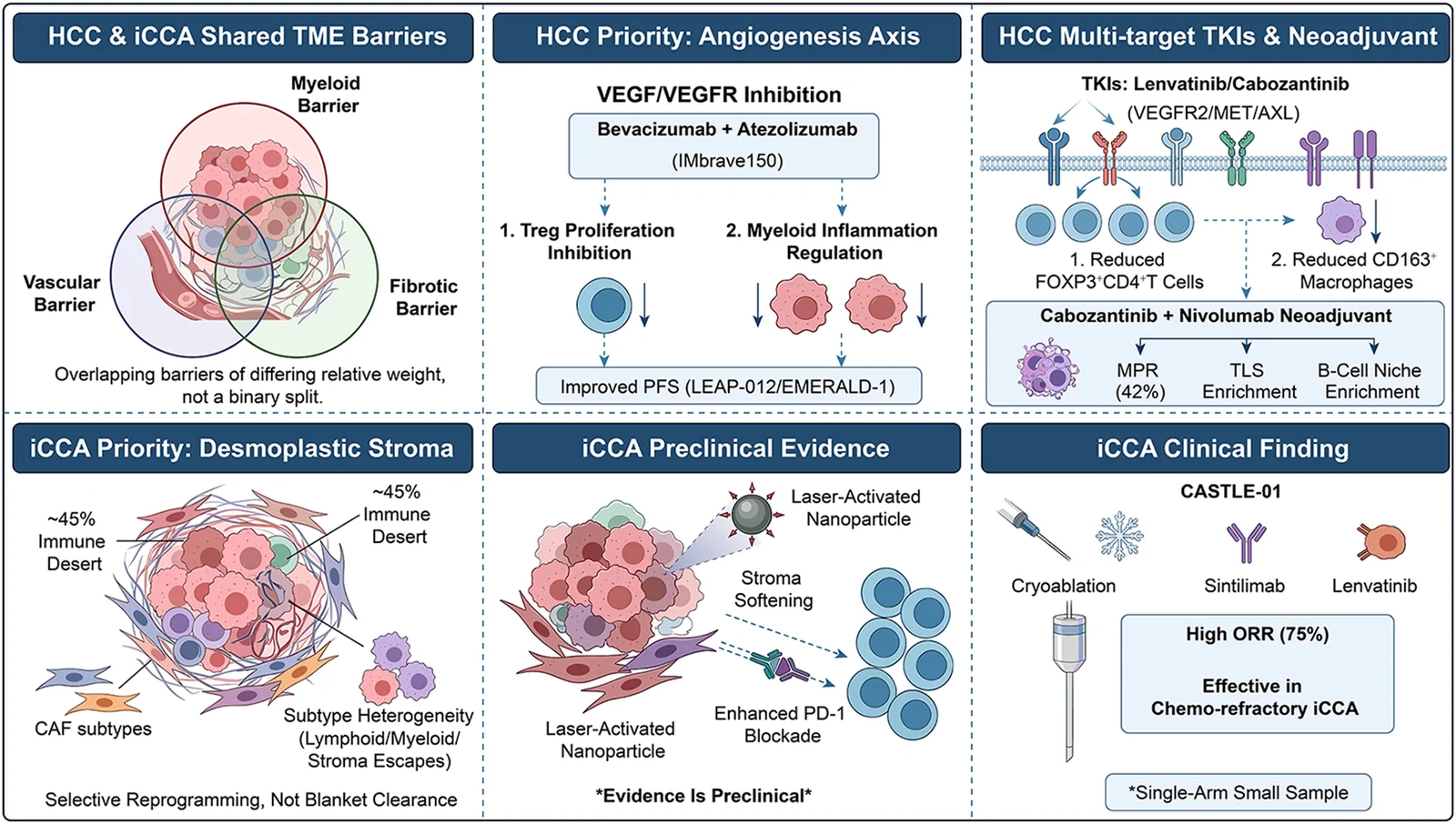
Overlapping barriers, distinct priorities: TME-targeted strategies for HCC and iCCA. The tumor microenvironments (TMEs) of HCC and iCCA share vascular, myeloid, and fibrotic barriers; their relative contributions differ among tumors but do not divide them into separate categories. Panels are arranged by tumor and by class of evidence, and are not intended to indicate a treatment sequence. In HCC, the angiogenesis-associated immunosuppression axis is a primary focus. VEGF/VEGFR blockade (e.g., atezolizumab plus bevacizumab in IMbrave150) inhibits Tregs and modulates myeloid inflammation. Multitarget TKIs (lenvatinib and cabozantinib) reduce specific immunosuppressive cell populations, and neoadjuvant combination therapy (cabozantinib plus nivolumab) has shown promising major pathological response (MPR) rates and enrichment of tertiary lymphoid structures (TLS). In iCCA, the focus shifts to the desmoplastic stroma and cancer-associated fibroblast (CAF) heterogeneity, with approximately half of tumors classified as immune deserts. Preclinical strategies explore stromal softening (e.g., laser-activated nanoparticles) to boost checkpoint blockade, while a clinical trial combining cryoablation, sintilimab, and lenvatinib showed a high objective response rate in a small cohort.

**Table 3:** Immunotherapy clinical trials — HCC vs iCCA, by mechanism-remodeling strategy*.

Tumor	Trial/phase	Regimen/setting	Mechanistic question	Key result	Interpretation/evidence limit	Ref
HCC	IMbrave150, phase III	Atezolizumab + bevacizumab vs sorafenib; first-line unresectable HCC	Checkpoint blockade plus VEGF-axis/vascular normalization	Median OS 19.2 vs 13.4 months; HR 0.66.	Practice-changing positive trial; bleeding/portal-hypertension risk affects eligibility.	(158)
HCC	CheckMate 9DW, phase III	Nivolumab + ipilimumab vs lenvatinib/sorafenib; first-line unresectable HCC	Dual PD-1/CTLA-4 blockade	Median OS 23.7 vs 20.6 months; HR 0.79; ORR 36% vs 13%.	High response durability, but early hazards and immune toxicity reinforce patient selection rather than a one-size-fits-all model.	(159)
HCC	COSMIC-312, phase III	Cabozantinib + atezolizumab vs sorafenib; first-line advanced HCC	PD-L1 blockade plus multikinase/VEGF targeting	PFS improved, but OS did not (16.5 vs 15.5 months; HR 0.98).	Key negative counterexample: mechanistic plausibility and PFS gain do not guarantee survival benefit.	(142)
HCC	HIMALAYA (STRIDE), phase III	Durvalumab + single-dose tremelimumab vs sorafenib; first-line unresectable HCC	Dual PD-L1/CTLA-4 blockade without anti-VEGF	Five-year OS 19.6% vs 9.4%; HR 0.76	Establishes a durable dual-ICI option; benefit is population-level and does not prove that HCC is uniformly exhaustion-dominant.	(141, 160)
BTC(includes iCCA)	TOPAZ-1, phase III	Durvalumab + gemcitabine/cisplatin vs chemotherapy; first-line advanced BTC	Chemotherapy-enabled priming plus PD-L1 blockade	Median OS 12.9 vs 11.3 months; HR 0.74; three-year OS 14.6% vs 6.9%.	Established standard, but the trial was powered for pooled BTC, not iCCA alone; avoid presenting the effect as iCCA-specific.	(145)
BTC(includes iCCA)	KEYNOTE-966, phase III	Pembrolizumab + gemcitabine/cisplatin vs chemotherapy; first-line advanced BTC	Chemotherapy plus PD-1 blockade	Median OS 12.7 vs 10.9 months; HR 0.83.	Confirms a modest class-level benefit; does not validate a particular stromal-remodeling mechanism.	(11, 161)
BTC(includes iCCA)	KEYNOTE-158, phase II	Pembrolizumab monotherapy; previously treated advanced BTC	Checkpoint release without mandated priming/remodeling	ORR 5.8%; median PFS 2.0 months and OS 7.4 months.	Useful negative benchmark for unselected monotherapy; rare biomarker-selected responders should be discussed separately.	(134)
iCCA only	CASTLE-01, phase II	Cryoablation followed by sintilimab + lenvatinib; previously treated advanced/metastatic iCCA	Local antigen release plus PD-1/VEGF-stromal modulation	n=28; ORR 75.0%, median PFS 16.8 months, OS 25.4 months.	Mechanistically informative and iCCA-specific, but single-arm, small and investigator-assessed; hypothesis-generating rather than practice-changing.	(157)

*Trials are grouped by the mechanism a regimen is designed to address, not by a demonstrated mechanism of benefit. All regimens listed administer checkpoint blockade concurrently with the partner intervention, with the exception of CASTLE-01, in which cryoablation preceded systemic therapy; that trial was single-arm and cannot distinguish an effect of the sequence from an effect of the components. No trial in either tumor has compared remodeling given before checkpoint blockade with remodeling given concurrently with checkpoint blockade. (4).

In iCCA, the emphasis shifts toward the desmoplastic stroma, although the relative prominence of this barrier varies across tumors. In one classification, approximately 45% of tumors were categorized as immune deserts, whereas lymphoid, myeloid, and mesenchymal subtypes exhibited distinct routes of immune evasion (16). Thus, stroma-directed strategies may be more appropriate for some tumors than for others, and CAF heterogeneity may favor selective reprogramming over indiscriminate depletion. iCCA-specific evidence remains largely preclinical (e.g., laser-activated nanoparticles designed to soften the matrix and enhance PD-1 blockade in CCA models (156). One clinical signal worth noting is CASTLE-01. In this single-arm phase II trial, 28 patients with chemorefractory advanced iCCA underwent cryoablation followed by sintilimab plus lenvatinib. The investigator-assessed ORR was 75.0% (95% CI 59%–91%), with cryoablated lesions excluded from response assessment. Paired multiomics analyses were consistent with increased immunogenicity and enhanced CD8^+^ T-cell penetration into tumor nests (157). Given the small sample size, single-arm design, lack of central review, and limited evidence in the post-first-line-standard setting, this study remains hypothesis-generating. It provides a proof-of-concept signal consistent with the possibility that barrier perturbation may provide a useful entry point into a shared suppressive network, but it does not establish that this treatment sequence is optimal or that any individual component drove the observed responses.

### 6.4 Spatial atlases and adoptive therapy

Spatially resolved multiplex assays may provide more predictive information than single-analyte biomarkers, although this evidence is derived primarily from heterogeneous pan-cancer data rather than prospective validation in HCC or iCCA. Across 8,135 patients and more than 10 tumor types, mIHC/IF achieved a pooled AUC of 0.79, compared with 0.65 for PD-L1 IHC, 0.65 for gene-expression profiling, and 0.69 for TMB (162). This difference is biologically consistent with, although not proven to result from, the ability of spatial assays to capture cellular coexpression and spatial arrangement; notably, only seven mIHC/IF analyses were included in the pooled analysis. Disease-specific studies are broadly consistent with this pattern but have not yet established a clinical hierarchy among these biomarkers. In HCC, an SPP1^+^-macrophage/CAF barrier at the tumor boundary was enriched in anti-PD-1 nonresponders, although the analysis included only eight treated patients (14). Separately, a tumor-intrinsic NRF2–COX2–PGE2 axis was retrospectively associated with resistance to atezolizumab plus bevacizumab, with plasma PGE2 proposed as a candidate biomarker that awaits prospective validation (163). In iCCA, immunosuppressive macrophage and hyperactivated Treg programs have been described as adverse-prognostic features, whereas intratumoral TLS abundance has been associated with better outcomes. Whether these features predict ICI-specific benefit rather than general prognosis remains unresolved (164).

Among cellular strategies, neoantigen-selected TILs provide one of the clearer early clinical signals, although not specifically in iCCA. In a single-arm phase II trial of treatment-refractory, mismatch-repair-proficient metastatic GI cancers (a mixed cohort that included cholangiocarcinoma), SEL-TIL therapy plus pembrolizumab produced an ORR of 23.5% (8/34; 95% CI 12.4%–40.0%), compared with 7.7% for SEL-TIL therapy alone. All patients also received lymphodepletion and high-dose IL-2 (165). Other approaches occupy different levels of evidence and should not be pooled. A personalized neoantigen DNA vaccine plus pembrolizumab achieved an ORR of 30.6% in HCC (166), GPC3-CAR-T combined with CD40 agonism remains preclinical (146), and PD-1/LAG-3 blockade has been evaluated only in an early-phase basket study containing a small, anatomically mixed cholangiocarcinoma cohort, such that iCCA-specific efficacy remains undefined (167). The broader lesson is sobering but clarifying: many reported iCCA-relevant cell-therapy signals derive from HCC or mixed GI cohorts, making iCCA-specific trials an important translational bottleneck rather than simply another mechanistic gap.

### 6.5 The therapeutic index

Intensive immune remodeling can be associated with mechanism-based risks, making dose, duration, and anatomical confinement potentially as crucial as pathway choice. STING agonism illustrates this point. In preclinical tumor models, low-dose intratumoral STING activation generated more durable tumor-specific CD8^+^ T-cell immunity than higher, ablative doses (168), suggesting that greater treatment intensity is not necessarily superior. The liver adds further caution: chronic endogenous STING activity promotes macrophage-mediated hepatic inflammation and fibrosis in NAFLD models (169). Importantly, for causal interpretation, hepatocyte ferroptosis generates oxidative DNA damage that *activates* macrophage cGAS–STING, rather than the reverse (170). These findings do not show that therapeutic STING agonists directly cause hepatocyte ferroptosis, but they caution that indiscriminate STING activation could aggravate inflammation in an already-diseased liver. Adoptive cell therapy poses a separate therapeutic-index challenge. In the neoantigen-TIL GI cancer trial, lymphodepletion was associated with transient severe hematologic toxicity, whereas high-dose IL-2 was associated with capillary leak and hemodynamic toxicity (165). Preclinical work suggests that continuous high-dose IL-2 can itself drive CD8^+^ dysfunction through STAT5–AhR signaling, although whether the short post-infusion IL-2 courses used clinically produce the same effect remains unproven (171). Modified TIL regimens that omit post-infusion systemic IL-2 have shown preliminary feasibility and manageable toxicity in early clinical studies outside liver cancer (172), and regional hepatic artery delivery is feasible in phase I studies of liver metastases (173). Neither approach, however, has yet demonstrated an improved therapeutic index in HCC or iCCA. For now, they should therefore be regarded as design considerations rather than validated solutions.

## 7 Discussion

Inadequate T-cell control in liver cancer arises from three distinguishable failure modes, and separating them clarifies what follows. Impaired priming constrains the number of tumor-reactive clones generated in the first place; exclusion then governs whether those clones ever reach malignant cells; and dysfunction governs what the cells that do arrive are capable of doing once there. Crucially, these processes operate in sequence rather than in parallel, so a defect introduced earlier in the chain cannot be rescued by an intervention acting further downstream. Checkpoint blockade, for instance, acts exclusively on the final step, meaning its ceiling is effectively set by the two steps that precede it. Nor are the three failure modes independent of one another: the desmoplastic matrix responsible for excluding T cells also generates the lactate and lipid gradients that subsequently restrain the cells that do enter, suggesting that exclusion and dysfunction may trace back to a shared origin, while myeloid populations recruited into the stromal compartment can simultaneously reinforce exclusion and blunt priming. We therefore treat the three not as alternative explanations but as differently weighted contributions within a single, interconnected network.

The architectural contrast between HCC and iCCA is best understood not as a difference in which failure mode operates, but as a difference in where along this sequence each tumor predominantly intervenes. In HCC, the vasculature, though abnormal, remains largely permissive, and the constraint is instead imposed on cells that have already arrived — through VEGF-driven regulatory T-cell expansion, impaired dendritic-cell maturation, and a lipid- and lactate-rich milieu that blunts the effector program — such that the characteristic lesion more often contains T cells that are present, antigen-experienced, and dysfunctional. In iCCA, by contrast, the barrier tends to act earlier: cross-linked collagen, elevated tissue stiffness, and CAF-derived CXCL12 more frequently retain T cells at the invasive margin and within the stroma before they ever reach the tumor-cell compartment. A cell that has never encountered antigen inside the tumor cannot, by definition, have been exhausted by it, so the deficit in this subset is positional rather than functional. These two modes are not mutually exclusive and likely coexist to varying degrees in both tumor types, but the predominant pattern carries two consequences worth emphasizing. First, it offers one explanation for why checkpoint blockade—which acts only at a step that presupposes both arrival and antigen encounter—tends to perform differently across the two tumors despite no difference in drug or target. Second, it implies that the most informative readout will differ by tumor type: the functional state of intratumoral T cells in HCC and their spatial position in iCCA. This leads to the testable prediction that a biomarker performing well in one setting may translate poorly to the other, a comparison that, to our knowledge, has not yet been made directly. The architectural descriptions themselves rest on human data in both tumors; the causal link between architecture and failure mode, however, is supported chiefly by preclinical intervention studies, and should be read as a proposed mechanism rather than a demonstrated one.

First, spatial position adds information beyond cell number. In iCCA, immune-cell localization across tumor-core, invasive-margin, and peritumoral compartments has been used to define spatial immunophenotypes associated with various clinical outcomes (45). However, these spatial categories are cohort- and sampling-dependent: a core biopsy may miss substantial regional heterogeneity, and an association between localization and outcome does not establish that localization is causally responsible for treatment failure. In liver tumors, SPP1^+^ macrophages interact with exhausted tumor-reactive CD8^+^ T cells within α-SMA-rich profibrotic regions (174), whereas separate HCC studies have implicated oncofetal endothelial states in the generation of immunosuppressive macrophages (175). These observations point to related mechanisms but do not establish a single causal cell triad, and their co-occurrence should not be interpreted as a universal causal circuit. The evidence instead supports a tendency rather than a rule: some HCCs contain intratumoral but functionally impaired T cells, whereas iCCA more often exhibits stromal, myeloid, or immune-desert states in which localization at the tumor margin may be particularly informative. These are cohort-level tendencies rather than fixed tumor-type laws. Integrating such cellular maps with MSI-based spatial metabolomics could localize lipid and selected metabolite gradients *in situ*, although sensitive lactate mapping still requires on-tissue derivatization (176) and remains subject to limitations in spatial coverage, analyte stability, and cross-platform standardization.

Second, monitoring should track the niche, not the genome alone. The principle worth adopting is spatially informed, longitudinal, multimodal monitoring that integrates circulating, biliary, and imaging signals to detect molecular or functional deterioration before radiographic progression. However, circulating and imaging signals may reflect tumor burden, treatment exposure, or variable tumor shedding as much as they reflect the immune state itself. Key unresolved questions are whether such signals can reliably predict deterioration and whether acting on them improves clinical outcomes; no prospective study in HCC or iCCA has yet answered both questions. Multimodal monitoring should therefore currently be viewed as a strategy for generating and refining hypotheses rather than as a validated decision rule.

Third, niche remodeling should be evaluated as a potential component of immunotherapy rather than assumed to precede it. Preclinical studies have provided biological plausibility but have not established a general stromal-first treatment paradigm. In preclinical iCCA, pan-LOX inhibition relieved vascular compression and, when combined with chemotherapy, was associated with stronger antitumor T-cell responses (33), whereas inhibition of the *ALOX5*–LTB4 axis reduced recruitment of immunosuppressive macrophages (177). Conversely, experience in other desmoplastic tumors, in which indiscriminate stromal depletion has failed to improve outcomes or may remove tumor-restraining functions, cautions against treating all stroma as uniformly pathogenic (178–180). Driver-matched clinical evidence remains far more limited. Pemigatinib combined with chemotherapy and pembrolizumab produced a complete metabolic response in a single case of *FGFR2*-fusion iCCA (181), whereas BET inhibition with JQ1 was active in preclinical IDH1-mutant/MYC-amplified iCCA resistant to the mutant-IDH1 inhibitor AG-120 (182). Neither a single case nor a preclinical response can establish the causal contribution of a specific component or justify a preferred treatment sequence. Taken together, these observations support a plausible but unproven hypothesis: in a subset of iCCA characterized by a measurable stromal–myeloid barrier, niche remodeling may improve T-cell access and enhance checkpoint activity. Whether this benefit is greater when remodeling precedes, rather than accompanies, checkpoint blockade remains to be determined, and no trial in either tumor has compared these sequences. Addressing this question would require a randomized, biomarker-stratified comparison of sequential versus concurrent administration, with patients grouped by *FGFR2/IDH1* genotype and CAF–myeloid spatial state and with paired on-treatment biopsies, designed so that the sequencing hypothesis is falsifiable. (4).

Underlying all three propositions is a constraint that should be stated plainly: the framework rests on evidence of uneven maturity. Some mechanisms are supported by direct human data, including spatial exclusion and margin sequestration of CD8^+^ T cells, desmoplastic barrier formation, myeloid and regulatory T-cell composition, and the FASN- and PCK1-associated metabolic programs; these carry our comparative claims most securely. Three components, however, are extrapolated in full. The precursor-exhausted reserve is defined in chronic viral infection and in transplantable tumor models; CXCL12–CXCR4 retention is established in pancreatic desmoplasia; and the metabolically restrained reserve proposed above rests wholly on non-iCCA systems and is advanced as a falsifiable hypothesis. That findings cannot automatically be transferred across tumor types is illustrated by CD39^+^PD-1^+^ CD8^+^ T cells, which are tumor-reactive in other malignancies but associated with adverse outcomes in iCCA. Provenance for each proposition is given in **Supplementary Table S2**; we therefore regard the extrapolated components as this review’s research agenda rather than its conclusions.

This framework should be considered a working model rather than a replacement for tumor classification: tumor type remains a useful prior, whereas the T-cell landscape may offer a more informative level of therapeutic refinement. The relevant question is therefore not only whether to release the checkpoint, but also which constraints predominate in an individual tumor and how the corresponding interventions should be combined or sequenced. The critical translational tests are whether these barriers can be measured reproducibly, whether they are causally actionable, and whether matching an intervention to the dominant barrier improves outcomes. Until these questions are answered, niche remodeling and other T-cell-directed combinations should be considered hypothesis-driven avenues for investigation rather than established treatment sequences.
