# Supplementary material for: Same organ, divergent immunity: T-cell exhaustion versus stromal exclusion in hepatocellular carcinoma and intrahepatic cholangiocarcinoma
Source: Front Immunol. 2026 Oct 16;17:1944650. doi: 10.3389/fimmu.2026.1944650 (PMC13720682; doi:10.3389/fimmu.2026.1944650)
Supplement: Supplementary file 1 [file Table1.docx]

**Supplementary Table S1 Molecular immune classifications of HCC and iCCA, with the authors' conceptual mapping onto a shared phenotype axis.**

Scope and comparability. The two panels present independently derived classifications based on different cohorts, platforms, clustering algorithms, and thresholds. Subtype frequencies are therefore reported within each classification and are not directly comparable across tumor types; no subtype in one panel corresponds to a subtype in the other. The shared phenotype axis in the left-hand column (immune-infiltrated, immune-altered, immune-excluded) is the conceptual alignment used in main text Table 1 to organize mechanistic discussion, and is a working model rather than a unified taxonomy.

Evidence category. All entries in both panels fall within category A (association) as defined in Supplementary Table S2, and derive from retrospective molecular and immune profiling of resected human tumor cohorts. No interventional, preclinical, or hypothesis-level content is included. Where a row lists an inferred mechanism, that mechanism is the one proposed by the source classification on associative grounds; causal support is preclinical rather than human and is assessed in main text Table 2 and Supplementary Table S2.

**Panel A. Immune classification of HCC**

| **Immune phenotype** | **HCC subtype** | **Freq.** | **Key molecular / immune features** | **Dominant T-cell state** |
| --- | --- | --- | --- | --- |
| **Immune-infiltrated** | Inflamed  (Immune +  Immune-like) | ~37%  (22%+15%) | • Molecular: high IFN signaling, CXCL9/CXCL10  • Immune: high cytotoxicity, diverse TCR, PD-L1+ | Well-infiltrated;  effector function preserved in the Active substate but dysfunctional in the Exhausted substate (~65% / ~35% of the Immune subclass) |
| **Immune-altered**  **(immunosuppressive)** | Intermediate | ~43% | • Molecular: enriched in TP53 mutations (49% vs 29%, p=0.035)  • Genomic: chromosomal losses of antigen-presentation / IFN genes (CIITA, CXCL9-11, IRF2)  • Inferred mechanism: transitional phenotype, no single dominant mechanism | Mild infiltration,  transitional, no single dominant mechanism (transitional) |
| **Immune-excluded** | Excluded | ~20% | • Molecular: enriched in CTNNB1 mutations (93% vs 27%, p<0.001); PTK2 overexpression (gene amplification / promoter hypomethylation)  • Immune: low PD-L1, low infiltration (“cold” tumor)  • Mechanism: WNT/β-catenin–mediated immune exclusion (impaired effector T-cell infiltration) | Excluded,  poorly infiltrated |

Subtype distribution: Inflamed 37% = Immune subclass 22% + Immune-like subclass 15%; Non-inflamed 63% = Intermediate 43% + Excluded 20% (Excluded, n=34/171)(19). Within the 22% Immune subclass, Active vs Exhausted subtypes derive from (18) (~65%/35% of the Immune subclass; the paper does not report a whole-cohort % for each, so this table does not assign them separate cohort-level frequencies).

CTNNB1 has a dual role, not a one-way biomarker: ~2/3 (69%) of WNT/β-catenin–activated tumours fall in the non-inflamed/Excluded class with immune paucity, but ~1/3 (31%) occur within the Inflamed class with strong interferon signaling and CD8+ infiltration — i.e. two distinct CTNNB1 patterns with opposite immune consequences(19).

**Panel B. Immune classification of iCCA**

| **Immune phenotype** | **iCCA subtype (STIM)** | **Freq.** | **Key molecular / immune features** | **Dominant T-cell state** |
| --- | --- | --- | --- | --- |
| **Immune-infiltrated** | Immune Classical | ~10% | • Molecular: canonical immune-activation pathways  • Immune: low desmoplasia | Well-infiltrated |
| **Immune-altered**  **(immunosuppressive)** | Inflammatory Stroma +  Hepatic Stem-Like | ~60%  (25%+35%) | **Inflammatory Stroma**  • Molecular: KRAS mutations (22% vs 8%, p=0.0006)  • Pathway: targetable oncogenic pathways (PI3K/AKT)  • Immune: T-cell exhaustion, abundant stroma  • Inferred mechanism: stroma-mediated exhaustion  **Hepatic Stem-Like**  • Molecular: BAP1 (28% vs 8%), IDH1/2 mutations; FGFR2 fusions (13% vs 5%)  • Pathway: stemness pathways NOTCH, YAP1  • Immune: cold tumor, ‘M2-like’ myeloid-rich  • Inferred mechanism: myeloid-driven suppression; YAP1-associated immunosuppression (not T-cell exhaustion) | Exhausted (Inflammatory Stroma)  Myeloid-suppressed (Hepatic-Stem-Like) |
| **Immune-excluded** | Tumor Classical + Desert-like | ~30%  (10%+20%) | **Tumor Classical**  • Naming: high cholangiocyte-marker expression (p<0.01)  • Molecular: TP53 mutations (26% vs 15%); TP53+KRAS co-mutation enriched (16% vs 4%, p=0.0003)  • Pathway: cell-cycle pathways  • Immune: non-inflamed “cold”, proliferation-driven; poor prognosis  **Desert-like**  • Molecular: Wnt/β-catenin signaling-driven  • Immune: lowest infiltration, Treg-rich (p<0.001, highest Treg positivity)  • Inferred mechanism: WNT/β-catenin–mediated immune exclusion | Proliferation-driven cold (Tumor Classical)  Near-complete desert (Desert-like) |

Subtype distribution: Inflamed 35% (Immune Classical 10% + Inflammatory Stroma 25%) / Non-inflamed 65% (Hepatic Stem-Like 35% + Tumor Classical 10% + Desert-like 20%). All percentages are of the whole iCCA cohort(20).Under the mapping used here, in which Inflammatory Stroma is placed in the immune-altered row, the immune-infiltrated fraction is 10% rather than the 35% inflamed fraction reported in the source.

Reassignment of Inflammatory Stroma. Inflammatory Stroma is grouped with Immune Classical in the inflamed category of the source classification. It is placed in the immune-altered row here because its dominant constraint is stroma-associated T-cell exhaustion rather than preserved effector function. The reassignment belongs to the conceptual mapping proposed in this review and does not reflect the source taxonomy.

Genotype features under Hepatic Stem-Like. STIM is transcriptome-based and does not correspond to duct-type anatomy; the genotype features listed for this class are enrichments within the class rather than defining assignments (20).

Abbreviations. HCC, hepatocellular carcinoma; iCCA, intrahepatic cholangiocarcinoma; IFN, interferon; PD-L1, programmed death-ligand 1; STIM, stroma–tumor–immune microenvironment classification; TCR, T-cell receptor; Treg, regulatory T cell. Gene symbols are italicized; protein and pathway names are not. Reference numbers follow the main manuscript.
