# Supplementary material for: Same organ, divergent immunity: T-cell exhaustion versus stromal exclusion in hepatocellular carcinoma and intrahepatic cholangiocarcinoma
Source: Front Immunol. 2026 Oct 16;17:1944650. doi: 10.3389/fimmu.2026.1944650 (PMC13720682; doi:10.3389/fimmu.2026.1944650)
Supplement: Supplementary file 2 [file Table2.docx]

Supplementary Table S2. Evidence provenance of the principal mechanistic propositions

Evidence categories: A, direct human evidence (patient specimens, cohorts, or clinical trials); B, indirect human evidence (cell lines, organoids, ex vivo tissue, or association without functional validation); C, preclinical models of HCC or iCCA; D, extrapolation from other tumour types or non-neoplastic disease, including chronic viral infection. “—” indicates that the proposition is not advanced for that tumour. Section and reference numbers follow the revised manuscript.

| Mechanism /proposition as presented in the review | Section | Strongest evidence in HCC | Strongest evidence in iCCA | Key References | Directly demonstrated in human iCCA? |
| --- | --- | --- | --- | --- | --- |
| Aberrant biliary repair and lineage plasticity establish the iCCA immune baseline (IL-33–ILC2–IL-13; Notch/AKT; Kras–AKT–mTORC1) | 2 | - | C | (21-25) | No. Derived entirely from murine biliary-injury and lineage-tracing models; the text states that whether this mechanism operates broadly in human iCCA remains unresolved. |
| CAF-driven collagen cross-linking (LOX family) and matrix stiffening act as a physical barrier to T-cell access | 2,4.1 | B | A(association)+C(causal reversal) | (26-33, 66) | Partly. CAF abundance, LOX-isoform upregulation and lesion stiffness are documented in human iCCA; reversal of the barrier and its immune consequences is shown only in orthotopic and spontaneous models (pan-LOX inhibition, ref 32). The text already notes that elastography gives bulk, lesion-level values that should not be equated with the cues individual cells sense. |
| Spatial topology, rather than lymphocyte density, governs exclusion; CD8⁺ cells accumulate at the invasive margin and tumour–stroma interface | 3.1,3.2 | A | A | (36-38, 43-45, 52, 58) | Yes. This is the best-supported iCCA-specific mechanism in the review, resting on human spatial transcriptomic, spatial single-cell proteomic and multiplex-imaging cohorts. |
| Tumour-intrinsic programmes drive exclusion in HCC (Wnt/β-catenin; SREBP1–monocyte axis) | 3.1 | A(association)+C(causality) | A (phenotype convergence only) | (18-20, 39-42) | Not applicable. Presented as an HCC mechanism; iCCA shares only the excluded phenotype at the Wnt-associated pole, not the demonstrated mechanism. The text now specifies that the Wnt and SREBP1 evidence is from preclinical and co-culture models. |
| IDH mutation and FGFR2 fusion shape antigen presentation and T-cell infiltration in small-duct iCCA | 3.1 | - | A (association) + C (mechanism) | (46-53) | Partly. Genotype–phenotype associations come from human cohorts; the IFN-γ–TET2 and cGAS-silencing mechanisms are model-derived, and clinical reversal has not been shown (ivosidenib-based combinations, ref 49). |
| Chronic antigen exposure drives a canonical CD8⁺ exhaustion programme (PD-1hi, TIM-3, TOX, TIGIT) that retains partial recoverability | 3.2 | A + B (ex vivo restoration) | B | (54, 57) | No. Human HCC specimens and ex vivo restoration support this in HCC; its operation in iCCA is inferred rather than directly shown. |
| IFN-γ–PD-L1 negative feedback is wired differently in space in the two tumours (malignant hepatocytes in HCC vs margin-resident myeloid cells in iCCA) | 3.2 | - | - | integrative inference; no primary citation | No. Explicitly presented in the text as an integrative inference from spatial and functional observations, not as an experimentally established pathway. Retained as a hypothesis. |
| Treg-mediated tolerance: MEOX1-associated hyperactivated Treg programme; TP53/KRAS co-mutation associated with Treg accumulation | 3.3 | - | A (association) + C (CD4 cytotoxic arm) | (20, 59, 60) | Partly. Human association is established; the intratumoural versus peritumoural distribution is inconsistent across cohorts, and the CD4⁺ cytotoxic programme derives from a murine cholangiocarcinoma model. |
| A reactive reserve exists (TCF1⁺ precursor-exhausted cells, CD69⁺CD103⁺ tissue-resident memory cells, tertiary lymphoid structures) that PD-1 blockade mobilises | 3.4,4.4,6.4 | B | D (TPEX) + B (TRM, TLS, descriptive) | (61-65, 77, 97-101) | No. The precursor-exhausted identity and its renewal capacity derive from chronic viral infection and transplantable tumour models; the CD69⁺CD103⁺ description in cholangiocarcinoma is human but descriptive and from a single cohort. The review itself documents a failure of cross-tumour transfer: CD39⁺PD-1⁺ cells mark tumour reactivity elsewhere but an exhausted-like, adverse phenotype in iCCA (ref 60 vs ref 61). |
| CXCL12–CXCR4 chemokine gradients retain effector T cells in CAF-rich regions | 4.1 | - | D | (69) | No. Directly established in pancreatic desmoplasia and described in the text as biologically plausible but not yet demonstrated in iCCA. |
| CAF–myeloid crosstalk builds a redundant suppressive network (FAP–STAT3–CCL2 → MDSC; GM-CSF-driven myelopoiesis; CSF1R blockade → CXCR2-dependent repopulation) | 4.1,4.3 | - | A + C (recruitment) / D (compensatory rerouting) | (70, 75, 76) | Partly. Myeloid recruitment is supported in patients and autochthonous models; the compensatory rerouting after CSF1R blockade, which motivates combination targeting, comes from other desmoplastic tumours and is explicitly stated to be untested in iCCA. |
| Bile acids reprogram the stromal and myeloid compartment (GPBAR1 on CAFs; arachidonic-acid transfer to macrophages via tunnelling nanotubes) | 4.2 | - | C | (73, 74) | No. Both supporting studies are iCCA-focused but preclinical. The widely assumed bile acid → TGR5 → suppressive TAM axis, and the c-Maf–IL-10/arginase programmes, are extrapolated from non-iCCA models and are flagged as unproven in the text. |
| Bile-acid signalling acts as a T-cell-intrinsic checkpoint (FXR–LAG3; reversal by UDCA) | 5.2 | C | B + C (human and murine iCCA; ex vivo human tumour fragments) | (91, 121-124) | Partly. This is the strongest human-adjacent iCCA-specific mechanism in the review, but the human component is ex vivo and the cohorts are small; no clinical data exist. Directionality is molecule- and cell-type-dependent, as the text notes. |
| Metabolic entrapment: a metabolically restrained, recall-competent T-cell reserve (AMPK–mTOR restraint; lactate at the Tcf7 super-enhancer; acidosis–SLC7A5–methionine) | 4.4 | - | D | (64, 77-85) | No. Every supporting observation derives from non-iCCA tumour models, chronic infection, or other malignancies, and the falsifying experiments proposed in this section are themselves adapted from other tumour types (TCR clonotype tracking in lung cancer, ref 76; lactate and pH perturbation, refs 77–79). The section is presented in its title and text as an explicitly falsifiable hypothesis, not as an established feature of iCCA. |
| Hypoxic and lipid-metabolic reprogramming suppresses T-cell function (HIF1A–APOE⁺ TAM; FASN programme; PCK1–LDHA–ferroptosis axis) | 4.5 | A/B | A (human iCCA single-cell and spatial profiling; 251-patient multi-omic cohort) + C (FASN inhibition plus anti-PD-1) | (93, 102-104, 107-116) | Yes for the HIF1A, FASN and PCK1 axes, which rest on human iCCA cohorts with model-based causal support. No for the absolute lactate and pH values (refs 100–102), which are cited for solid tumours generally and are not measured in iCCA; the text already labels these as contextual. |
| Extracellular vesicles extend immune and stromal remodelling beyond the tumour cell (exosomal miR-183-5p → TAM PD-L1) | 5.1 | - | B | (89, 117-120) | No. Studies are largely cell-line-based or descriptive; CAF-EV-induced T-cell anergy and TLR-dependent EV exclusion are explicitly identified in the text as unsupported. Near-term value framed as biomarker rather than mechanism. |
| Desmoplasia and regional heterogeneity constrain the interpretation of liquid biopsy | 5.3 | A | A (heterogeneity, low shedding) + B (bile cfDNA) | (45, 52, 125-132) | Partly. Regional immune heterogeneity and low plasma ctDNA shedding are documented in human CCA; bile-derived cfDNA evidence comes from invasively sampled biliary-stricture cohorts rather than mass-forming iCCA, as the text states. |
| Defective antigen presentation and cDC1 loss are a proximal barrier, at least as limiting as inhibitory signalling | 6.1 | A/B | C | (13, 96, 136-140) | No. Progressive cDC1 loss is documented in experimental iCCA only; the therapeutic corollary (APC activation plus PD-1 blockade) rests on preclinical models of both tumours. |
| Adding a second, positive immune input (OX40, 4-1BB, ICOS, CD40 agonism; STING agonism) overcomes what checkpoint blockade alone cannot | 6.2 | C | C | (146-148) | No. No clinical efficacy has been demonstrated in iCCA or in randomised studies; the sotigalimab data are in vitro. Presented as rationale for investigation. |
| Therapeutic priorities differ by subtype: vascular-axis remodelling in HCC versus stroma-directed remodelling in iCCA | 6.3 | A (randomised trials plus on-treatment human biopsies) | A (CASTLE-01, single-arm, n = 28) + C (nanoparticle stromal softening) | (141-145, 149-157, 159-161) | Partly. The HCC arm is anchored in randomised trials with correlative human tissue. The iCCA arm rests on one small single-arm investigator-assessed trial plus preclinical work; the review already labels it hypothesis-generating. |
| Spatially resolved multiplex assays outperform single-analyte biomarkers; neoantigen-selected cell therapy is a viable route | 6.4 | B | D | (14, 162-166) | No. The biomarker comparison is a pooled pan-cancer meta-analysis (8,135 patients, only seven mIHC/IF analyses); the cell-therapy signal comes from a mixed GI cohort that included cholangiocarcinoma. iCCA-specific validation is absent, as the section concludes. |
| Intensity, duration and anatomical confinement of immune remodelling determine the therapeutic index | 6.5 | C | C/D | (165, 168-173) | No. Dose–response for STING, hepatic STING–fibrosis biology, and IL-2/lymphodepletion toxicity derive from models and from trials conducted outside liver cancer. |
